# Supplementary material for: Machine learning for syndromic surveillance using veterinary necropsy reports
Source: PLoS One. 2020 Feb 5;15(2):e0228105. doi: 10.1371/journal.pone.0228105 (PMC7001958; doi:10.1371/journal.pone.0228105)
Supplement: S1 Text — (DOCX) [file pone.0228105.s001.docx]

**S1 Text. Supplemental Tables.**

**Table A. Optimal Hyperparameters for Algorithms Using TF-IDF Features.**

| **Model** | **Parameter** | **Best Values** | **Mean** | **SD** |
| --- | --- | --- | --- | --- |
| ***Logistic Regression*** | Cost | 100, 1000, 1000, 100, 10, 10, 100, 100, 10, 10 | 244 | 380 |
| ***Support Vector Machine*** | Cost | 2048, 8, 8, 512, 32, 8, 32, 8, 512, 128 | 330 | 604 |
|  | Gamma | 0.0078125, 0.125, 0.125, 0.03125, 0.125, 0.125, 0.03125, 0.125, 0.03125, 0.125 | 0.085 | 0.049 |
|  | Kernel type | Radial basis function for all folds | N/A | N/A |
| ***Classification Tree*** | *min_samples_* | 5, 10, 10, 2, 10, 50, 10, 2, 2, 5 | 10.6 | 13.6 |
| ***Bagging Trees*** | None | N/A | N/A | N/A |
| ***Random Forest*** | *m* | 0.75*p*, 0.5*p*, 0.2*p*, 0.5*p*, 0.2*p*, 0.1*p*, 0.2*p*, 1.0*p*, 0.1*p*, 1.0*p*  (for feature space of size *p*) | 0.455^a^ | 0.336^a^ |
|  | *min_samples_* | 5, 2, 2, 2, 2, 2, 2, 2, 5, 5 | 2.90 | 1.37 |
| ***Gradient Tree Boosting*** | Learning rate | 0.1, 0.01, 1.0, 0.01, 0.01, 1.0, 0.001, 0.01, 0.01, 0.01 | 0.216 | 0.392 |
|  | Maximum tree depth | 7, 7, 8, 10, 7, 8, 10, 5, 10, 9 | 8.10 | 1.58 |

For each model hyperparameter included in the grid search, the best values are given for each fold of 10-fold external cross-validation. For each hyperparameter combination, performance was measured by an F1 score computed across 10 internal cross-validation folds.

^a^ Mean and standard deviation describe the distribution of coefficients of *p*.

**Table B. Performance Metrics for Algorithms Using Unigram and Bigram TF-IDF Features.**

|  | **GI Disease** | | **Respiratory Disease** | | **Urinary Disease** | |
| --- | --- | --- | --- | --- | --- | --- |
| **Model** | **Accuracy** | **F1** | **Accuracy** | **F1** | **Accuracy** | **F1** |
| Logistic Regression | 0.870  (0.850, 0.889) | 0.876  (0.855, 0.897) | 0.881  (0.862, 0.901) | 0.871  (0.847, 0.893) | 0.919  (0.902, 0.935) | 0.580  (0.494, 0.663) |
| Support Vector Machine | 0.876  (0.855, 0.896) | 0.882  (0.859, 0.901) | 0.890  (0.871, 0.909) | 0.877  (0.853, 0.899) | 0.944  (0.929, 0.958) | 0.691  (0.613, 0.764) |
| Classification Tree | 0.906  (0.887, 0.923) | 0.906  (0.886, 0.924) | 0.963  (0.950, 0.975) | 0.959  (0.945, 0.972) | 0.967  (0.956, 0.978) | 0.841  (0.788, 0.892) |
| Bagging Trees | 0.926  (0.909, 0.941) | 0.926  (0.908, 0.941) | 0.964  (0.952, 0.975) | 0.961  (0.947, 0.972) | 0.971  (0.960, 0.981) | 0.856  (0.796, 0.906) |
| Random Forest | 0.918  (0.901, 0.934) | 0.918  (0.901, 0.934) | 0.962  (0.949, 0.973) | 0.958  (0.944, 0.972) | 0.976  (0.966, 0.985) | 0.881  (0.832, 0.925) |
| Gradient Tree Boosting | 0.910  (0.892, 0.927) | 0.909  (0.890, 0.927) | 0.967  (0.956, 0.978) | 0.964  (0.951, 0.975) | 0.971  (0.961, 0.981) | 0.857  (0.804, 0.904) |

Three syndrome classification tasks (gastrointestinal, respiratory, and urinary) were tested, using both unigram and bigram features that appear in the corpus more than once. Accuracy and F1 scores were assessed by 10-fold cross-validation, with 95% confidence intervals in parentheses calculated by bootstrapping.

**Table C. False Negatives in the Random Forest Model.**

| **Error Type** | **GI** | **Respiratory** | **Urinary** | **Total** |
| --- | --- | --- | --- | --- |
| ***Unusual terms*** | 23 %  (11/47) | 38 %  (6/16) | N/A^a^ | 27 %  (17/63) |
| ***Species-specific anatomical descriptors*** | 28 %  (13/47) | 13 %  (2/16) | 0 %  (0/21) | 18 %  (15/84) |
| ***Terms derived from causative organisms*** | 23 %  (11/47) | 6 %  (1/16) | 0 %  (0/21) | 14 %  (12/84) |

Several types of false negatives for the random forest model were classified and quantified by manual review. The categories were mutually exclusive except for four documents shared across two categories.

^a^ Given the smaller prevalence of urinary pathology in the training dataset, our assessment was that it was not possible to characterize unusual terms for the urinary disease syndrome.

**Table D. Examples of Terms Associated with False Negative Error Types in the Random Forest Model.**

| **Error Type** | **GI** | **Respiratory** |
| --- | --- | --- |
| ***Unusual terms*** | lip, tongue, glossitis, cholangitis, sinusoidal, Peyer’s, GALT, typhlitis, cecitis | pleuritis, mediastinum, tracheitis, laryngotracheitis, BALT, sinusitis |
| ***Species-specific anatomical descriptors*** | proventriculus, coelomitis, cloacal, beak, crop, gizzard, yolk, rumenitis, abomasal, abomasum, reticulum | sacculitis |
| ***Terms derived from causative organisms*** | toxocara, cryptosporidium, cryptosporidial, strongyle, clostridiosis, coccidiosis, coronaviral, coronavirus | gallid |
