# Supplementary material for: Machine learning for syndromic surveillance using veterinary necropsy reports
Source: PLoS One. 2020 Feb 5;15(2):e0228105. doi: 10.1371/journal.pone.0228105 (PMC7001958; doi:10.1371/journal.pone.0228105)
Supplement: Attachment — Submitted filename: Response to Reviewers.docx [file pone.0228105.s002.docx]

Dear Dr. Pławiak and reviewers,

In this letter, I will address each of the points raised in your review. I have reproduced your points in bold.

Comments to the Academic Editor

**Please ensure that your manuscript meets PLOS ONE's style requirements, including those for file naming.**

To the best of my knowledge, all applicable style requirements have been followed in the revised manuscript.

**We note that Figure 8 in your submission contains [map/satellite] images which may be copyrighted… We require you to either (1) present written permission from the copyright holder to publish these figures specifically under the CC BY 4.0 license, or (2) remove the figures from your submission.**

Since Google Maps does not respond to requests for explicit permission, we have decided to remove this image from the manuscript.

**If there are ethical or legal restrictions on sharing a de-identified data set, please explain them in detail (e.g., data contain potentially identifying or sensitive patient information) and who has imposed them (e.g., an ethics committee). Please also provide contact information for a data access committee, ethics committee, or other institutional body to which data requests may be sent.**

We are unable to publish a de-identified data set because the relevant information consists of free-text laboratory reports. The Wisconsin Veterinary Diagnostic Laboratory (WVDL) has an ethical responsibility to protect the confidentiality of test results and reports. Further, requirement 4.1.4.c of the American Association of Veterinary Laboratory Diagnosticians’ (AAVLD) accreditation requirements mandates that the WVDL have standard operating procedures to protect confidential client information, including test results. Please note that the data under consideration do not serve a commercial purpose for this institution.

In accordance with this ethical responsibility and the AAVLD requirement, WVDL policy allows for a researcher to contact a laboratory staff member or quality manager (Kristin Zuzek, 1521 E. Guy Ave, Barron WI, 54812). The researcher should complete a Third Party Information Request Form, which will then be forwarded to the WVDL director (Keith Poulsen, 445 Easterday Lane, Madison WI, 53706) for final review.

Comments to Reviewer #1: Major Comments

**The exact counts for each of the classes should be specified, not just the percentage in the dataset.**

The manuscript was edited to state exact counts.

**The bootstrapped cross-validation procedure should be described in the manuscript. It is not a standard methodology that readers may not be familiar with.**

The manuscript was edited to describe this procedure.

**The use of unigrams limits the performance of the Logistic Regression and the SVM models. I believe the Logistic Regression model---or an SVM with a linear kernel (check LinearSVC in sklearn)---would perform better if both tfidf-weighted unigrams and bigrams are included.**

The manuscript was edited to explain that the inclusion of bigrams did not significantly increase the performance of the classifiers studied in this paper, which included an L2-regularized logistic regression model. This data was added to the supplemental text.

**The LSTM results should be included in Table 1. Otherwise, it is cumbersome to compare Table 4 with Table 2.**

My interpretation of your comment is that the data in Table 4 should be integrated into Table 2 (Table 1 contains example phrases and is not related to the data in tables 2 and 4). In the revised manuscript, I have merged these two tables.

Comments to Reviewer #1: Minor Comments

**While out of scope for this paper, it would be interesting to know if pretraining neural network-based models on non-veterinarian electronic medical records (e.g., MIMIC) could improve its performance.**

We are also interested in how the performance in this task might be affected by pretraining on related data sets but decided that this analysis was not in the scope of our paper. Considering the relevance of public human clinical databases, it is worth noting that veterinary pathologic text contains many terms that are unique to animal anatomy and disease.

**The data availability statement should be mentioned in the manuscript.**

My understanding from the Academic Editor’s feedback is that the data-availability statement will be updated based on our clarifications of the ethical and regulatory issues affecting the publication of these free-text medical reports. I would be happy to include a comment about data availability in the body of the manuscript if doing so is appropriate, but I am concerned that this would be redundant. Academic Editor**:** If applicable, can you please provide guidance on what information regarding data availability should be included in the body of the manuscript?

Comments to Reviewer #2: Minor Comments

**Lines 95,96 combining the morphologic findings and final diagnosis sections or, if both of those were empty, by combining all sections (15% of cases).**

**Have you tried any other ways of dealing with empty data elements?**

No obvious alternatives were available to deal with reports for which the morphologic or final diagnosis sections were blank. Our approach acknowledged that the remaining sections of the reports may contain valuable data but placed a clear priority on utilizing the morphologic or final diagnosis sections if they were present, as was the case in most documents. Since the empty data elements in question were entire sections of free-text diagnostic reports, please note that there would have been no meaningful way to impute the missing information.

**Line 109 “Phrases from Wisconsin Veterinary Diagnostic Laboratory (WVDL) necropsy reports that were judged by a human reviewer as representing gastrointestinal, respiratory, or urinary pathology.”**

**Perhaps more than one human reviewer should provide the judgements.**

The main purpose of this table is to provide the reader with text examples that clearly describe the indicated syndrome. The caption was updated to reflect the fact that both of our expert pathologist reviewers agreed on the characterization of these text examples.

**Line 113 “Two veterinarians board-certified by the American College of Veterinary Pathologists reviewed the 1,000 documents and classified each as having evidence of GI disease and/or respiratory disease and/or urinary disease based on clinical experience.”**

**Having more than two veterinarians involved in the study would result in a more reliable review.**

Our primary aim in this paper was to study the suitability of machine learning models for classifying necropsy reports according to the represented syndromes. To achieve this aim, it is reasonable to accept an individual expert’s judgement as a ground truth for model training so long as the expert is consistent with other experts on a subset of the data. This was the approach taken in our study. We utilized two expert annotators who are board-certified specialists in the American College of Veterinary Pathologists and established that they agreed on 97.2% of cases.

The cost of additional human reviewers is often a significant barrier in this type of study. As mentioned in Line 425, additional research may focus on techniques specifically designed to address the limited availability of expert labels.

**Line 438 “This approach could also help uncover baseline trends in case numbers. “**

**Line 77 “…automated approach to syndromic 78 surveillance within animal populations.”**

**The data used in the study was obtained from one center: Wisconsin Veterinary Diagnostic Laboratory (WVDL) at the 86 University of Wisconsin-Madison. The use of data from one center in the case of generalization suggests that there may exist certain trends in the way the necropsy reports are prepared by veterinarians. Using data from more than one veterinary laboratory would provide a more appropriate basis for generalizing the results.**

The manuscript has been edited to emphasize that we did not attempt to generalize beyond the WVDL case population.

All authors have approved the submission of this revised manuscript and supplemental text. Thank you for your consideration of our work.

Your sincerely,

Nathan Bollig, DVM

Postdoctoral Trainee, Comparative Biomedical Sciences Training Program, School of Veterinary Medicine

*and*

Ph.D. student, Computer Sciences

University of Wisconsin
4720 Medical Sciences Center
1300 University Avenue
Madison, Wisconsin 53706

Phone: (608) 728-4280

Email: nbollig@wisc.edu
